# Single‐cell RNA sequencing in osteoarthritis

**DOI:** 10.1111/cpr.13517

**Published:** 2023-06-14

**Authors:** Yuyuan Gu, Yan Hu, Hao Zhang, Sicheng Wang, Ke Xu, Jiacan Su

**Affiliations:** ^1^ Institute of Translational Medicine Shanghai University Shanghai China; ^2^ Organoid Research Center Shanghai University Shanghai China; ^3^ School of Medicine Shanghai University Shanghai China; ^4^ Department of Orthopedics Shanghai Zhongye Hospital Shanghai China; ^5^ Wenzhou Institute of Shanghai University Wenzhou China

## Abstract

Osteoarthritis is a progressive and heterogeneous joint disease with complex pathogenesis. The various phenotypes associated with each patient suggest that better subgrouping of tissues associated with genotypes in different phases of osteoarthritis may provide new insights into the onset and progression of the disease. Recently, single‐cell RNA sequencing was used to describe osteoarthritis pathogenesis on a high‐resolution view surpassing traditional technologies. Herein, this review summarizes the microstructural changes in articular cartilage, meniscus, synovium and subchondral bone that are mainly due to crosstalk amongst chondrocytes, osteoblasts, fibroblasts and endothelial cells during osteoarthritis progression. Next, we focus on the promising targets discovered by single‐cell RNA sequencing and its potential applications in target drugs and tissue engineering. Additionally, the limited amount of research on the evaluation of bone‐related biomaterials is reviewed. Based on the pre‐clinical findings, we elaborate on the potential clinical values of single‐cell RNA sequencing for the therapeutic strategies of osteoarthritis. Finally, a perspective on the future development of patient‐centred medicine for osteoarthritis therapy combining other single‐cell multi‐omics technologies is discussed. This review will provide new insights into osteoarthritis pathogenesis on a cellular level and the field of applications of single‐cell RNA sequencing in personalized therapeutics for osteoarthritis in the future.

## Graphical abstract

Schematic of the applications of scRNA‐seq in OA. Single‐cell RNA sequencing could elucidate the molecular mechanisms in osteoarthritis and identify potential targets to design targeted therapies. scRNA‐seq can also evaluate the safety and effectiveness of therapeutics by clarifying the cell population changes in the surrounding microenvironment. In the future, combining multiple single‐cell multiomics technologies will further pave the way for personalized treatment.

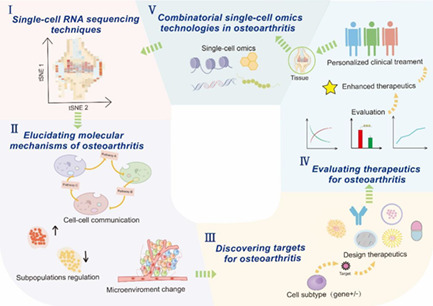

## 1 INTRODUCTION

Osteoarthritis (OA) is a progressive and degenerative disease of joints, resulting in pain, stiffness, and functional limitation. Risk factors mainly include age (30% of individuals greater than 45 years old have radiographic evidence of knee OA), followed by the female sex, obesity, genetics and joint injury.^1^ The prevalence of OA is rising due to the growing aging population and the obesity epidemic^2^ with 250 million people worldwide currently affected, becoming a major public health challenge in the coming years.^3^

OA is usually characterized by pathological features such as cartilage destruction, subchondral bone remodelling, synovitis, and meniscus degeneration, which ultimately leads to joint deformity.^4^, ^5^ Additionally, as a heterogeneous disease with different clinical phenotypes that continuously evolve, OA patients eventually guide to common clinical manifestations making classification of phenotypes of OA difficult in clinical studies.^6^ Currently, scientific research on revealing OA mechanisms mainly focused on typical pathological structural alternations, and several studies have demonstrated that genotype changes might be related to the subtle microenvironment changes resulting in clinical phenotypes changes. For example, by integrating RNA sequencing data of tissues from OA patients, Yuan et al. exemplified a classification model that links the molecular functions of genes relating to clinical symptoms of OA subtypes to discover the relationship between genotype and clinical phenotype of the disease.^4^ Despite efforts over the past decades to develop the understanding of novel molecular mechanisms, however, they are not enough to fully elucidate OA pathogenesis. On the other hand, conventional RNA sequencing technologies can only provide the average gene expression signals emitting from an ensemble of cells, thus, hiding the heterogeneous signals from specific genes from a single cell. Recent evidence demonstrated that even in similar cell types, genetic level signals can be heterogeneous.^7^ Therefore, using an effective method to better understand the mechanisms in the onset and progression of OA is of critical importance.

Since the invention of single‐cell RNA sequencing technology (scRNA‐seq), some defects can be filled.^8^, ^9^ The importance of single‐cell analysis for understanding disease development and progression has been widely applied in studies such as heterogeneity in tumours.^10^ Using single‐cell technology to identify the primary clone or subclones in the lineage made it easier to fully construct tumour lineage heterogeneity.^11^ As a powerful strategy to associate genotypes with phenotypes,^12^ scRNA‐seq reveals rare cell populations in an organism,^13^ builds cell–cell communication networks^14^ and recreates the trajectories of different cell subpopulations during development.^15^ Up to now, single‐cell can reveal part of the cell atlas including cartilage, synovium, meniscus and subchondral bone, and it is difficult to address the stiff bones partly due to the issues relating to limited isolating approaches of bone cells. Thereby, a better understanding of bone metabolism and development at a higher resolution level contributes to elucidating the mechanisms of the pathogenesis of bone diseases. However, data provided by scRNA‐seq are difficult to relate to the disease phenotype of each patient, researchers are unable to design suitable treatments for each patient. With combined single‐cell technologies, based on the clearer mechanisms of the interactions of cell subclusters revealed by scRNA‐seq, bone biologists still need more effort to adapt single‐cell approaches to solving particular challenges.^16^

In a word, the diagnosis of OA is usually established late in the disease process, therefore, too late to expect much help from drugs to modify OA.^2^, ^17^ Thus, identifying substantial biochemical markers^18^ or molecular mechanisms offers potential for diagnosis, intervention and new treatments for OA.^19^ With its unique high‐resolution view of cellular transition, scRNA‐seq can reliably describe OA progression on a cellular level, discovering targets to design better treatment methods and evaluating the effectiveness of therapeutics. Finally, classifying patients according to their prognosis paves the road to individualized therapy for OA.

In this paper, we reviewed recent research on the applications of scRNA‐seq in OA for the first time, briefly introducing the general principles of scRNA‐seq. Then, the findings in different structural tissues highlight the discovery of potential pathogenesis and targets in OA. Finally, we discussed and nominated that, combined with other single‐cell omics techniques, scRNA‐seq will contribute to the deeper interpretation of mechanisms and evaluate promising personalized therapeutics for OA in the future.

## 2 SINGLE‐CELL RNA SEQUENCING TECHNIQUES

**FIGURE 1:**
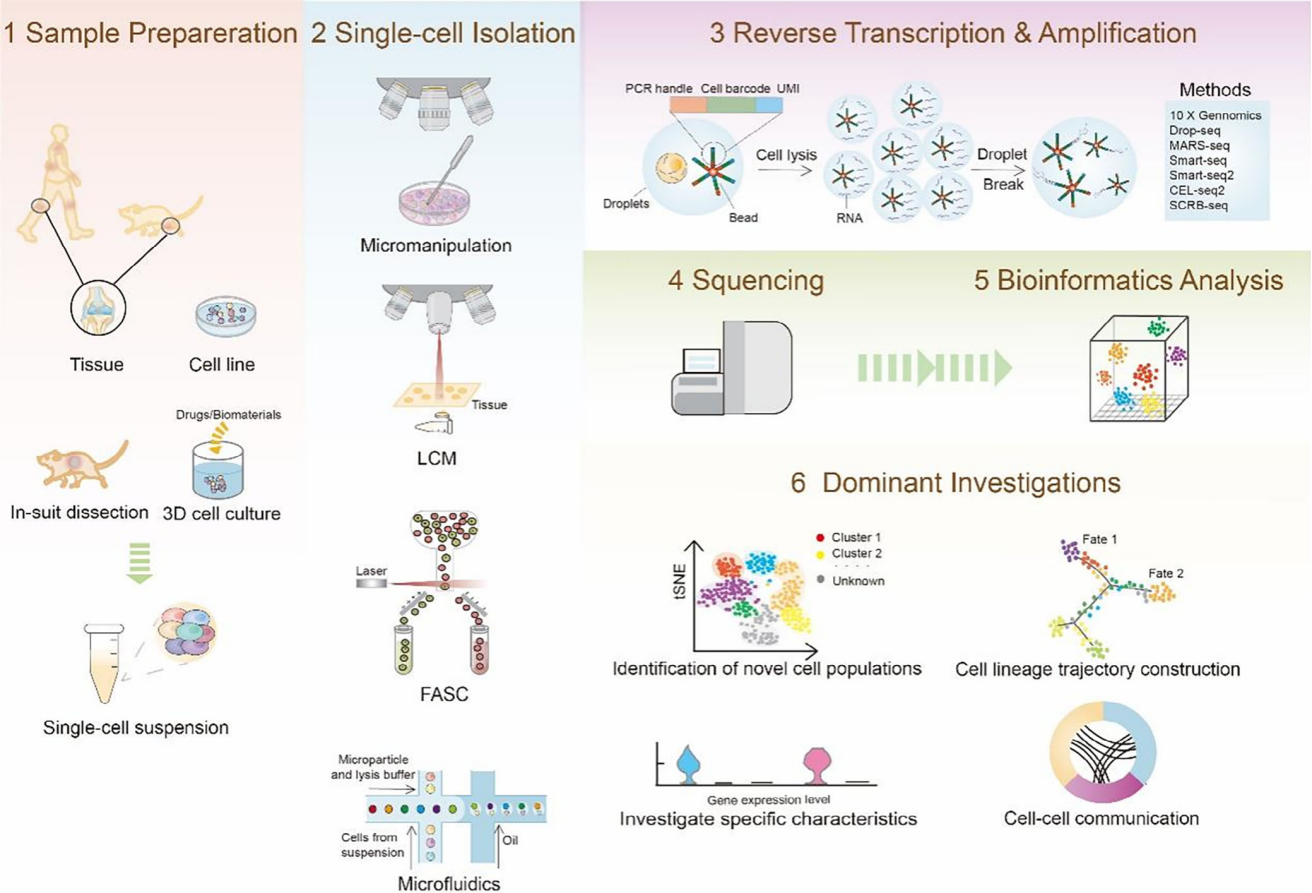
Workflow of scRNA‐seq. Single‐cell RNA sequencing technology prepares single‐cell suspensions by isolating single‐cell from tissues or cell lines co‐culturing with biomaterials/drugs. Then, establishing gene libraries, and performing bioinformatic analysis of sequencing results to identify new cell subpopulations, lineage tracing and cell–cell communication. FACS, flow‐activated cell sorting; LCM, laser capture microdissection.

Before scRNA‐seq was widely used, it is rather challenging to identify cell surface markers that are neither unknown with prior knowledge of genes or proteins nor characterized by multiparametric flow‐activated cell sorting (FACS) analysis or large‐scale cytometry.^20^ However, scRNA‐seq enabled an unbiased, alternative workflow that has made it more appropriate to sequence the transcriptome of cells when elucidating the composition of cell types, even if starting with small amounts of material.^21^, ^22^ All single‐cell RNA‐seq protocols share a common workflow^23^ (Figure 1): (1) sample preparation; (2) single‐cell isolation; (3) reverse transcription and amplification (including library preparation); (4) sequencing; (5) bioinformatic analysis.

As a prerequisite to generating robust data sets, sample preparation should be optimized depending on sets of characteristics of samples, for example, tissue or cell types and culture conditions, such as 3D organoid models.^24^ Inherent perturbations in samples dissociating into a single cell also make it difficult to interpret scRNA‐seq data. When addressing bone marrow stromal or calvarial osteoblast cultures, changes in these extremely heterogeneous cells may become unable to distribute distinct phenotypes observed in vitro, confounding experimental interpretations.^16^ Tissue dissociating into single‐cell is typically conducted enzymatically at 37 °C under gentle mechanical agitation. In this step, the appropriate proteolytic enzyme and the duration of digestion limit the excessive cell lysis and maximize the yield of single‐cell by decreasing the number of dead cells.^23^, ^25^ However, single‐nucleus RNA sequencing (snRNA‐seq) was applied to address snap‐frozen samples to overcome some artefacts caused by the single‐cell dissociation. Additionally, several instrumental methods were adopted to ensure the purity and integrity of single cells, avoiding incomplete enzymatic digestion and contaminations such as cell doublets DNA or RNA.^26^

Instrumental methods to isolate single cells vary in the number of cells (high‐throughput or low‐throughput) and the selection (biased or unbiased).^27^ Micromanipulation is a time‐consuming and low throughput method that utilizes microscope‐guided capillary pipettes and extracts single cells labelled with markers from a suspension.^28^ Nevertheless, cellular integrity is maintained, which is workable with rare cells in particular. Laser capture microdissection (LCM) can isolate tissues laid on a thermoplastic polymer film with heat‐activated infrared or ultraviolet laser aided by computer systems. It is rapid that carries out isolation omitting solid samples dissociation and is utilized in isolating chondrocytes from growth plate cartilage for RNA isolation.^29^ LCM can also preserve positional information of each cell from frozen tissues for spatial transcriptomic profiling. FACS depends on antibody affinity to cells labelled with specific surface markers that are already known and run through flow cytometry increasing the specificity and sensitivity when addressing a bulk sample.^30^ FACS can clear doublets or dead cells and directly assesses population frequencies of interest populations. This method omitting additional enrichment steps is eligible for bone biology because MSCs are frequently outnumbered by haematopoietic cells in specimens, avoiding unwanted erythroid lineage populations captured.^31^, ^32^, ^33^ But the potential limitations include the requirement for large starting volumes (>10,000 cells) and the signal overlaps from large samples may affect the isolating purity. Other bone cell types, such as osteocytes, are also challenging to dissociate.

Microfluidic technology with precise fluid control gradually gained popularity due to its low sample consumption and cost. Recently, microfluidic technologies had been wildly applied in the latest methods in upgrading single‐cell technologies. μCB‐seq (Microfluidic Cell Barcoding and Sequencing) combining high‐resolution imaging system enabled to preload of primers inside addressable reaction chambers of a microfluidic device to improve gene detection sensitivity.^34^ However, different approaches in microfluidic technologies have various advantages in global increasing the capture efficiency, compared to plate‐based approaches that give rise to additional zero values which make it difficult to tell apart from a biological variation of interest.^35^ In hydrodynamic trap approaches of this technology, they passively isolate based on cell size and allow for long‐term cell culture for further experiments such as drug treatment.^36^ One of the commercial platforms using hydrodynamic trap approaches, Fluidigm C1, is based on a nanoliter automatic microfluidic instrument that offers lower false positives causing less bias than tube‐based technologies but is limited by homogeneous cell sizes.^37^ In droplet‐based approaches, they embedded every single cell in a hydrophilic droplet suspending hydrophobic channels to make it appropriate to isolate rare cell types with high throughput and yield. Another promising approach of microfluidic technology is microdroplet‐based devices that allow the mono dispersion of aqueous droplets in a continuous oil phase and cell barcodes also be added within the droplet to distinct different cell origins.^38^ Equipped with microdroplet‐based microfluidics, the commercial Chromium system from 10× Genomics allows the profiling of simultaneously prepared single‐cell libraries in a heterogeneous biological space. However, because of the frequency bias of different cell types, mature multinucleated osteocytes may physically disrupt or otherwise lose in the microfluidic isolating step.^39^ Although some types of bone cells with harsh isolation procedures, single‐cell ATAC‐seq (scATAC‐seq) multiplexing with scRNA‐seq methods could provide a more settled way to assess cell states to suit requirements.^40^

Largely determined by scRNA‐seq protocols, the sequencing depth is a significant experimental parameter to be considered when separating complex tissue at hand, as well as experimental objectives. These protocols have strengths and weaknesses in linear amplification, length coverage, low copy RNA species detection, high throughputs, and cost reduction. Six commonly used methods to synthesize cDNA from single‐cell mRNA/RNA: cell expression by linear amplification and sequencing 2 (CEL‐seq2), Drop‐seq, massively parallel RNA single‐cell sequencing (MARS‐seq), SCRB‐seq, Smart‐seq and Smart‐seq2.

CEL‐seq2 utilized linear strand‐specific transcript amplification mainly at 3′ ends reducing mRNA molecule counting biases with unique molecular identifiers (UMIs), a short unique sequence.^8^ The introduction of UMIs brings about a lot of advantages by integrating into cDNAs before amplification allows for the identification of clustering as normalization and reduces nonlinear PCR amplification bias. However, once cell‐type heterogeneity is solved with clustering and lineage analysis, unwanted noises are introduced while most drop‐outs disappear. Heterogeneity‐inspired pre‐processing tool (HIPPO) is more flexible to leverage zero proportions for low UMI data sets from 10× protocols.^41^

Single cell RNA barcoding and sequencing (SCRB‐seq) is a high throughput protocol with 12,000 cells per time and also includes UMIs. A significant advancement was made with Smart‐seq that full‐length cDNA synthesis could complete about 40% of transcripts in 2012, followed by Smart‐seq2 which was updated later in 2013.^42^ Smart‐seq has laid the foundation for the remarkable development of scRNA‐seq technologies employing stable template switching ribs (guanosine) 3 oligos and enhancing recovery rates of low expression genes. MARS‐seq can separate and sort 100–1000 cells into individual wells by using fluorescence‐activated cell sorting. This index sorting protocol allows for the identification of unique cell types across different model systems and organisms.

Drop‐seq and InDrop‐seq are similar methods in the throughput. The main difference between them is that Drop‐seq uses reagent‐containing beads while the latter uses reagent‐carrying hydrogel microspheres.^12^ 10× Chromium also has similarities with the two previous methods that employ a gel bead in the emulsion microfluidic capture method but can process eight samples at once. Overall, each method has different advantages in the amplification step, Smart‐seq2 can detect the most numerous genes per cell, while other microfluidic methods display less amplification noise when using the UMIs.^43^ In addition, the sensitivity was sequencing depth‐dependent. The 10× Genomics Chromium protocol was maturely developed and delivered a high degree of sensitivity and accuracy in scRNA‐seq and is suited to most experimental research.^44^

Four key steps are typically involved in the data analysis process: data cleaning and normalization, dimension reduction, clustering and post‐clustering examination. Dimension reduction is performed after reading count normalization to avoid ‘the curse of dimensionality’. Principal component analysis is an essential method for unsupervised linear dimensionality reduction but consumes a long computation time for large‐scale scRNA‐seq datasets. A more effective strategy is that iteratively conduct the data analysis process with different numbers of principal components (PCs). For example, using populations relating to chondrocytes, osteoblasts, or other already delineated cell populations as ‘landmarks’ to aid in internal control.^16^ t‐SNE (t‐distributed stochastic neighbour embedding) is implemented in the popular Cell Ranger pipeline (10× Genomics) to visualize samples and the Seurat R package is an additional non‐linear reduction method that is widely used to project cells into 2D space.^21^

Cell‐type identification is a dominant feature of scRNA‐seq. Recently, many algorithms have been developed and assessed to attain cell clustering and cell‐type identification quickly and accurately.^45^ One of these studies compared predicted and observed cell types and proportions to identify the best tool for synovium.^46^ Reconstructing dynamic cellular trajectories in different stages of the disease concerning differentiation or cell cycle progression was also developed to reveal the microenvironment changes with time. ‘Pseudotime’ in Monocle tools can measure the biological progression of cell subclusters, but this notion is different from ‘real‐time’ because cells are sampled all at once. However, for those cells including endosteal and periosteal mesenchymal cells, the pseudotime analysis assumption may be false.^32^ In addition, RNA velocity, a concept based on the different ratios between unspliced and spliced RNA when a gene is being up‐ or downregulated in the cell differentiation process, describes the direction and speed automatically, such as scVelo analysis. Cell linkages can examine gene regulatory systems contributing to the disease process by combining gene or protein regulatory networks for deep phenotype interactome analysis.^47^ Cell–cell communication based on platforms, Seurat and Scanpy, have been developed to complete most analysis steps. To sum up, some commercial companies have provided software tools for data analysis, but its infancy remains to test and gold‐standard tools have yet to be developed to obtain an agreement.

Available technologies for scRNA‐seq have unique strengths and weaknesses. Researchers are supposed to consider the scale of the experiment, the sensitivity of each method and the biological question to be answered, before the research begins.

## 3 ELUCIDATING MOLECULAR MECHANISMS OF OSTEOARTHRITIS

**FIGURE 2:**
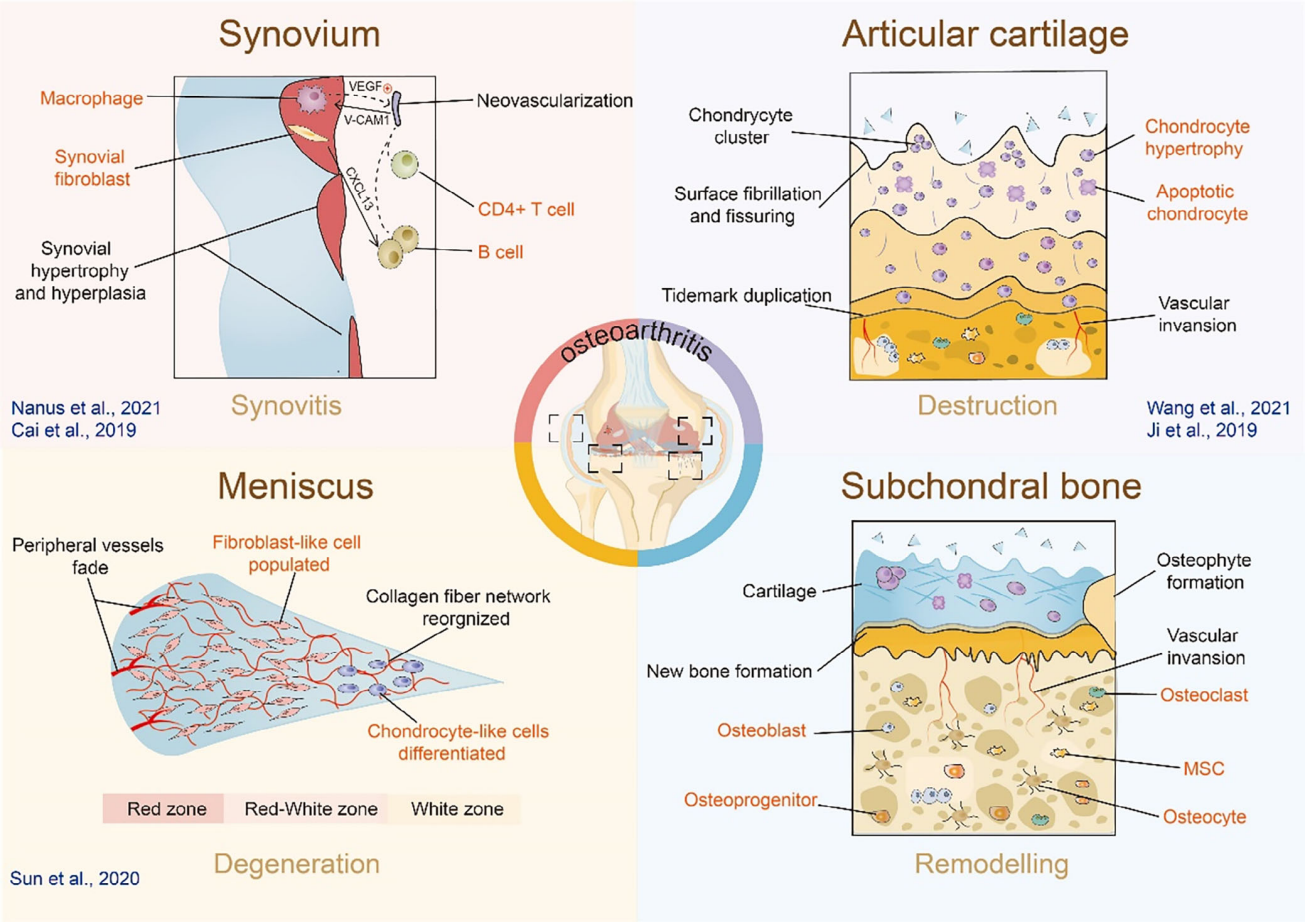
Selected scRNA‐seq studies revealing OA heterogeneity. Osteoarthritis involves pathologic structure changes including articular cartilage destruction, subchondral bone remodelling, meniscus degeneration, and synovitis. Recently, single‐cell RNA‐sequencing has been performed to reveal the molecular mechanisms of microenvironmental alterations in these sites. The transformation of cellular states may relate to the irreversible deterioration process in damaged structures leading to OA progression.

The data of scRNA‐seq opens the door to answering the biological questions in OA: what cell types exist within high molecular heterogeneous diseased tissues? How to do cellular state switches work to manifest OA physiologic derangements? How do gene expression correlations regulate OA progression?^48^ A comprehensive understanding of the pathogenesis relies on elucidating mechanisms with heterogeneous symptoms, including articular cartilage destruction, subchondral bone remodelling, synovitis and meniscus degeneration.^5^, ^49^ (Figure 2) Next, we will describe the current applications of scRNA‐seq in the study of the molecular mechanisms of OA (Table 1).

**TABLE 1:** Summarizing the most important candidates in each tissue of joint.

Tissue structure	Cell subcluster	Cell type	Pathogenic mechanism	Key related gene/cytokines/protein	Reference
Articular cartilage	ECs (effector chondrocytes)	Chondrocytes	Energy supply in chondrocytes and regulate the metabolism process in OA progression	CYTL1	[53]
	RegCs (regulatory chondrocytes)		Regulate many signalling pathways and interact with immune cells	CHI3L1	
	HomCs (homeostatic chondrocytes)		Modulating homeostasis of articular cartilage and regulate circadian clock in OA progression		
	ProC (proliferative chondrocytes)		Affect RNA metabolic processes and RNA stabilisation		
	preHTC (prehypertrophic chondrocytes)		Biological adhesion and multicellular organismal process as a hypertrophic chondrocyte phenotype		
	HTC (hypertrophic chondrocytes)		Promotes chondrocytes apoptosis and calcium deposition to modulate mineralization of surrounding matrix		
	CPCs (cartilage progenitor cells)		Stem‐cell‐like progenitor cells and migrate toward damaged cartilage tissue with repair ability	BIRC5, CENPU, UNE2C, DHFR and STMN1	
	FCs (fibrocartilage chondrocytes)		Promote cartilage fibrosis and vascularization		
	pre‐FCs (pre‐fibrocartilage chondrocytes)		Collagen fibril organization, cell adhesion and migration, and vasculature development, existing in the late‐stage OA	COL1A1	[70]
	Fibroblastic‐like chondrocytes		Involved in the ECM‐receptor interaction pathway and promoted human OA chondrocyte apoptosis	ACTG1; COL6A3	[72]
	RepCs (reparative chondrocytes)		High reparative ability		
	StrCs (stressed chondrocytes)		Regulate ferroptosis in cartilage damage of OA	HSPB1; GPX4	[76]
Synovium	End‐stage OA, pain	Synovial fibroblasts (SFs)	Shifted toward a more succinct fibroblast pathotype in OA progression	HTRA3; GPX3	[85]
	Early‐stage OA, pain		Promote nociceptive signalling pathways	KCNMA1; ACKR4	
	Early‐stage OA, no pain		Relate to synovial hyperplasia	FN1; GSN	
	SFs in state 5		Release chemokines and pro‐inflammatory factors	VCAM1 and TLRs	[91]
	SFs in state 2		Expressed genes similar to End‐stage OA, and pain		
	Activated fibroblasts		Maintain ECM		[95]
	Immune cell recruiters		Activation of leukocytes	CHI3L1	
	Barrier macrophage subclusters	Synovial macrophages	Inhibit inflammation with a tight‐junction‐mediated shield for intra‐articular structures	CX3CR1	[106]
Meniscus	FCPs (fibrochondrocyte progenitors)	Chondrocyte‐like cells	Differentiate into various cell lineages	CD146	[100]
	DegPs (degenerated meniscus progenitors)		Recruitment of meniscus progenitor cells	CD318	
	FCs (fibrochondrocytes)		Contribute to cartilage homeostasis in the physiological status	COL1A1	
	ECs progenitors	Endothelial cells	Generate vessels to maintain a blood supply, but also promote the migration of meniscus cells.		[106]
	ECs subclusters		Express intrinsic apoptosis factors, CASP3	HOXA13	
	ECs subclusters		Express intrinsic apoptosis factors, BID	RASGRF2	
Subchondral bone	Pre‐ECs (precursor‐endothelial cells)	Endothelial cells	Mediating inflammation pathways and exosome anabolism to participate in the crosstalk between chondrocytes and endothelial cells		[113]
	ECs (endothelial cells in subchondral bone)		Promote angiogenesis but also be coupled with OBs		
	EnOBs (Endothelial osteoblasts)	Osteoblasts	Correlated with OBs proliferation	MGST1; VCAM1	
	StOBs (Stromal osteoblasts)		Associated with collagen fibril organization	Col11A1; GFPT2	
	MinOBs (Mineralised osteoblasts)		Relate to ECM mineralization	WIF1; BGLAP	

### 3.1 Articular cartilage

Articular cartilage is the most important part of the knee joint and is vulnerable to the outside changes. Articular cartilage destruction due to injury or mechanical stimuli,^50^ aging and other risk factors commonly contribute to the development of OA. Despite being composited of singular cell type–chondrocyte, the cartilage used to lack a detailed understanding of the internal state and phenotypic changes of chondrocytes.^51^ scRNA‐seq opens doors for novel interpretation.

**FIGURE 3:**
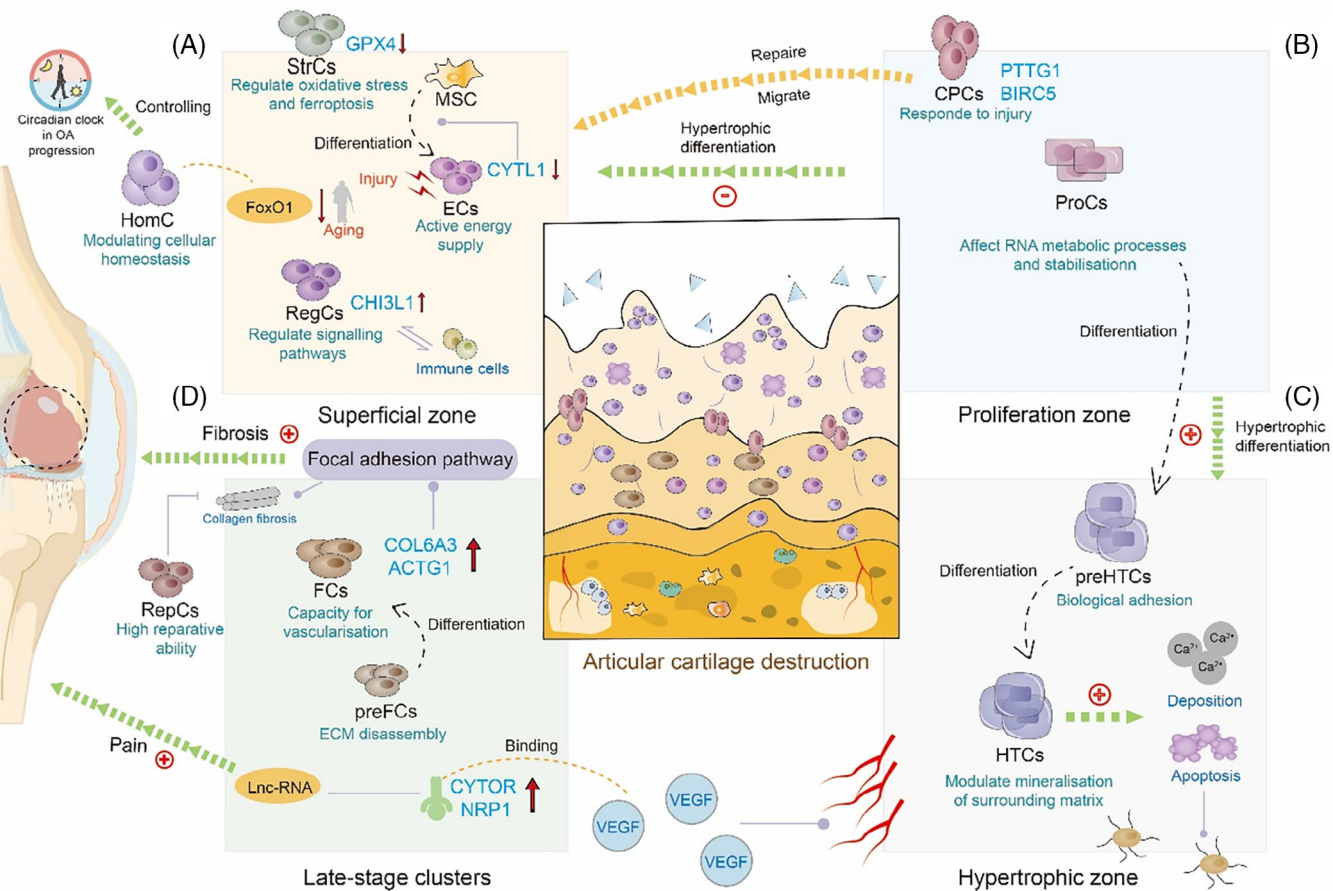
Applications of scRNA‐seq in articular cartilage. Single‐cell RNA sequencing reveals the progression of articular cartilage in human osteoarthritis. (A) ECs down‐regulated CYTL1 and RegCs up‐regulated CHI3L1 resulted in disorders of cartilage homeostasis in superficial zone. StrCs highly expressed GPX4 to interfere the cartilage self‐repair mechanisms. (B) ProCs are flat and columnar chondrocytes in the proliferative zone. The top layer of ProCs has the potential to prevent hypertrophic differentiation while the lower layers close to preHTCs and HTCs allow hypertrophic differentiation. (C) preHTCs and HTCs control the hypertrophic differentiation in hypertrophic zones and ECM organization during the progression of human OA. (D) In end‐stage OA, preFCs and FCs promote the fibrosis process in OA. Repair cells such as HomCs, RegCs and CPCs offer clues for OA cartilage regeneration.

Differentiated from BMSC, chondrocytes were found to be heterogeneous in the previous studies,^52^ which formed into three regions (superficial zone, proliferation zone, hypertrophic zone) of articular cartilage. Following the chondrogenesis, chondrocytes produce a collagenous extracellular matrix (ECM) to reduce the friction and the impacts on the joints, but in the OA progression, the chondrocytes began to change into fibrosis and hypertrophic phenotypes, followed by the degradation of ECM, which was shown in the fibrillation and fissuring of the cartilage surface. Ji et al. has identified new subtypes of chondrocytes and signalling pathways involved in OA pathogenesis based on scRNA‐seq analysis to reveal the internal states of chondrocytes.^53^ Three novel clusters were identified: effector chondrocytes (ECs), regulatory chondrocytes (RegCs) and homeostatic chondrocytes (HomCs). ECs, active in energy supply during the development of chondrocytes,^54^ are characterized by cytokine‐like 1 (CYTL1) that has been shown to promote chondrogenic differentiation from MSCs (mesenchymal stem cells).^55^, ^56^ Sebastian et al. also found that CYTL1 was down‐regulated after injury negatively affecting cartilage degeneration using scRNA‐seq data analysis.^57^ RegCs phenotype may regulate many signalling pathways during OA progression. A small proportion of RegCs, enriching for B cell and T cell receptor signalling, might play a role in the reaction with immune system. Another study showed RegC, marked with highly expressed human cartilage glycoprotein chitinase 3‐like‐1 (CHI3L1), was markedly expanded in OA cartilage,^58^ supporting that CHI3L1 can be a potential marker for staging the severity and progression of OA^59^, ^60^ (Figure 3A).

Chondrocyte hypertrophy and matrix lesions will give rise to the occurrence of cartilage destruction in OA.^61^, ^62^, ^63^ Ji et al. further defined proliferative chondrocytes (ProC), prehypertrophic chondrocytes (preHTC) and HTC (hypertrophic chondrocytes) populations with specific markers and a potential transiting relationship amongst them were also investigated (Figure 3B) The switch from the proliferating phenotype–ProCs to the more active hypertrophic phenotype–HTCs promotes chondrocytes apoptosis and calcium deposition, ultimately attracting vascular and bone‐cell invasion^53^ (Figure 3C). During the hypertrophic progression of chondrocytes, runt‐related transcription factor‐2 (RUNX2) was further activated to regulate chondrocyte phenotype alternation by inducing ECM mineralization and vascular invasion, thereby many ECM proteases such as matrix metalloproteinase‐13 (MMP13) have accelerated cartilage collapse and OA progression.^61^ In the gene expression pattern predicted by scRNA‐seq data, NRP1 co‐expressed with CYTOR as a receptor for a vascular endothelial growth factor (VEGF), sponging with lncRNA^64^ to further induce angiogenesis, which contributes to structural damage of cartilage and pain in OA progression.^65^, ^66^ Hypertrophic chondrocytes can also produce mineralised ECM proteins and leave small cavities within the tissue after apoptosis, giving enough room for channel formation to enable cell passage between cartilage and subchondral bone, leading to the loss of the articular cartilage in late‐stage OA.^67^

The cartilage ECM further destroyed its structure and deteriorated its biomechanical properties and metabolic homeostasis in late‐stage OA.^68^ During this period, the de‐differentiation of chondrocytes induces the increased expression of a fibrotic gene such as collagen type I.^69^ Ji et al. have identified fibrocartilage chondrocytes (FCs) that are associated with cartilage fibrosis. Chou et al. also discovered a new cluster–pre‐fibrocartilage chondrocytes (pre‐FCs) with similar biomarkers to FCs. Both FCs and preFCs (preFCs) highly expressed the fibroblast‐related marker gene, COL1A1, and were enrichment of genes associated with collagen fibril organization, cell adhesion and migration and vasculature development.^70^ Li et al. defined a cluster of fibroblastic‐like chondrocytes that up‐regulated actin gamma 1 (ACTG1), which was involved in the ECM‐receptor interaction pathway and promoted human OA chondrocyte apoptosis.^71^ Furthermore, up‐regulated COL6A3 also relates to collagen fibrosis.^72^ The GSEA and KEGG analyses also suggest that up‐regulated ACTG1 and COL6A3 might participate in the pathological process of OA through the focal adhesion pathway, which may affect ECM composition and abnormal changes in OA osteocyte phenotypic^73^, ^74^ (Figure 3D). Taken together, ECs distributed at the start of the chondrocyte trajectory and FCs mainly at the end of the trajectory, indicating the switch of the chondrocyte phenotypes from the vulnerable healthy types to the OA phenotypes. Along the trajectory, the multi‐chondrocyte phenotypes will be the potential candidates to regulate the OA progression.

Especially in the damaged areas of chondrocyte ECM, chondrocyte subclusters may destroy cartilage homeostasis to accelerate OA progression. Lv et al. analysed the damaged area of human articular cartilage tissues and found a subcluster related to ferroptosis in cartilage damage of OA. The stressed chondrocytes (StrCs) with high frequency in the damaged area of OA, preferentially expressed HSPB1 (heat shock protein B1).^75^ StrCs were sorted into four subclusters (C1‐1 to C1‐4). Among them, C1‐3‐4 (a subcluster of C1‐3) expressing higher levels of ferroptosis marker genes than apoptosis, such as GPX4 (glutathione peroxidase 4), interfered with the cartilage self‐repair mechanisms. The experimental results of mouse cartilage showed that GPX4 positively correlated with TRPV1 (transient receptor potential vanilloid 1), which regulate together to protect chondrocytes from ferroptosis during OA progression.^76^

Although lacking intrinsic reparative ability, articular cartilage still contains some cell populations that might be involved in the maintenance of homeostasis. Ji et al. found that HomCs with highly expressed genes of human circadian clock rhythm markers and favourable genes against OA, showed the ability to control the circadian clock in OA progression.^77^ Sebastian et al. found that FoxO1, as a downstream mediator of TGF‐β, was significantly reduced in aging mice, showing the ability to regulate articular cartilage autophagy and homeostasis.^57^, ^78^, ^79^ Chou et al. also identified a subcluster–reparative chondrocytes indicate a high reparative ability.^70^ The so‐called cartilage progenitor cells (CPCs) have already been identified and characterized that expressed stem‐cell‐related surface markers (BIRC5, CENPU, UNE2C, DHFR and STMN1).^80^, ^81^ scRNA‐seq help to reveal the origin and functional properties of CPCs and specifically discovered its markers for further study. Additionally, CPCs were also found to be migratory toward damaged cartilage tissue from the middle and deep zones to the superficial zone.^58^

### 3.2 Synovium

**FIGURE 4:**
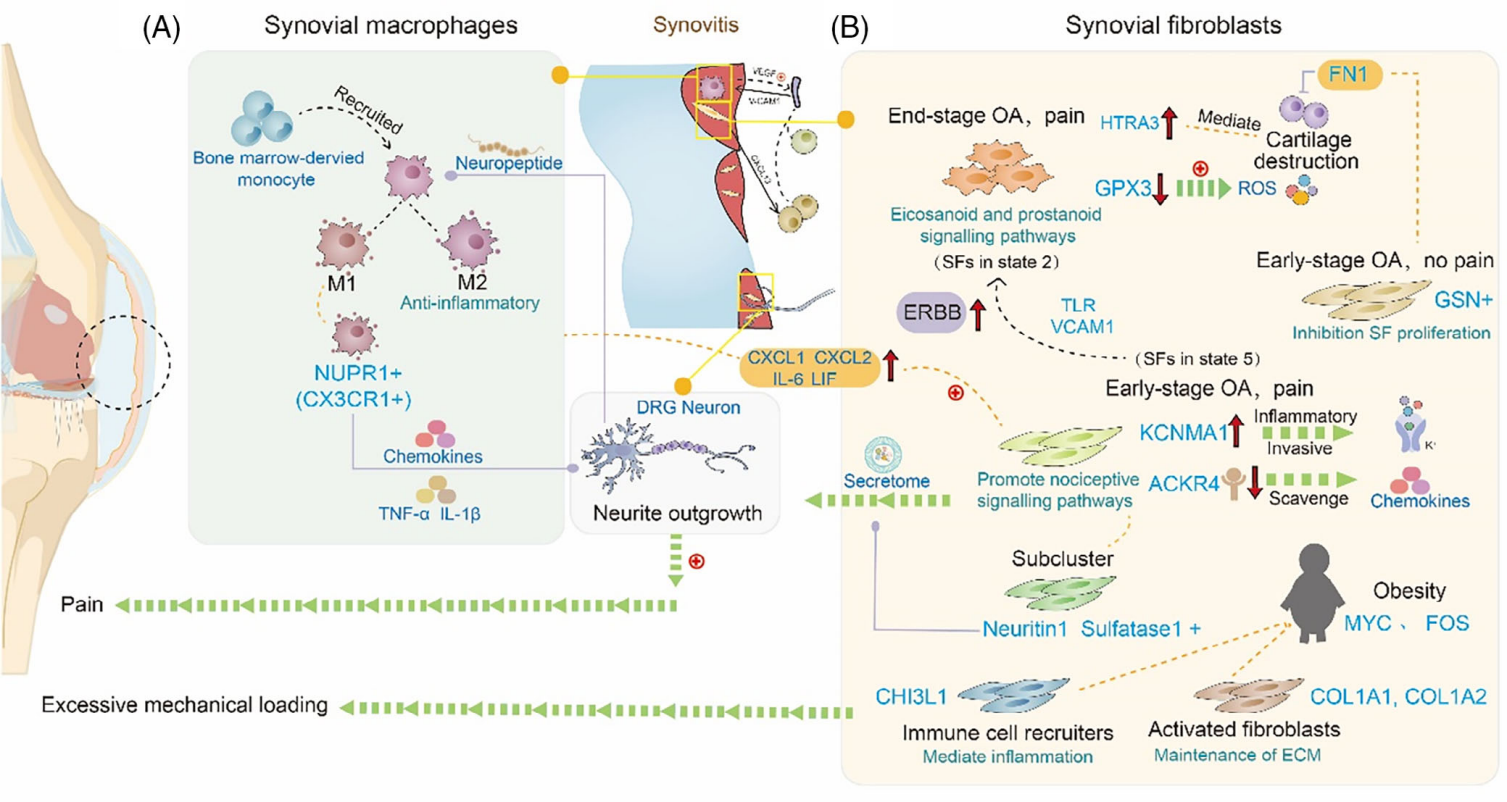
Application of scRNA‐seq in synovium. (A) Synovial macrophages showed special subtypes releasing inflammation mediators to aggravate OA pain. (B) Synovial fibroblasts exhibited different phenotypes from sites of joint pain in knee OA patients. Obese OA patients with ‘activated fibroblasts’ and ‘immune cell recruiters’ subclusters to activate synovitis by excessive mechanical loading.

The structural changes in synovium are mainly associated with the symptoms, such as pain and synovitis, during OA progression. Synovitis encompasses a range of abnormalities, such as fibrosis, infiltration of macrophages and synovial lining hyperplasia.^82^ Synovitis presents early at pre‐radiographic stages in OA and is typified by hyperplasia of synovial fibroblasts (SFs) appearing to increase with advancing structural deterioration. scRNA‐seq has been performed on the functional states of SFs to discuss whether any impact of the transition states of SFs on synovitis will be the spoilers at different OA stages.^83^ (Figure 4B).

Synovial tissue exhibited hypertrophy and hyperplasia in the end‐stage OA with different phenotypes in patient‐reported pain sites and synovitis may appear before the cartilage degeneration in the early stage OA. With distinct SF subsets in the early‐ and late‐stage OA, apparently, patterns of synovitis are closely related to these SFs subsets, which has been identified the heterogeneous of SF in rheumatoid arthritis (RA).^84^ Also, the interaction with immune cells is another factor to affect OA. Nanus et al. have identified different subclusters of SFs with either early‐stage or end‐stage knee OA using scRNA‐seq.^85^ Compared to early‐stage OA patients, end‐stage OA patients have a greater degree of synovitis. Additionally, the differential pain degree of synovial tissue may extend to the cell state alternations of the OA SFs. The end‐stage OA with the majority of one large cluster (end‐stage OA, pain) suggests that OA progression shifted toward a more succinct fibroblast pathotype. This cluster, highly expressed HTRA3, may relate to ECM degradation meditating cartilage destruction and GPX3 against oxidative damage was down‐regulated in the expression level. The painful synovial tissue sites in early stage OA patients (early‐stage OA, pain) contain subclusters of highly expressed genes that early stage OA, pain, including CXCL1, CXCL2, IL‐6 and LIF. Meanwhile, there are subclusters with increased KCNMA1, involved in regulating pro‐inflammatory and pro‐invasive properties by opening calcium‐sensitive potassium ion channels and decreased ACKR4 functioning to scavenge chemokines.^86^, ^87^ These data further accounted for the relationship that SFs in painful synovial sites played an important role in synovial inflammation during the development of early‐stage OA pain. A specific subcluster exhibited high expression of neuritin 1 and sulfatase 1. Neuritin 1 is regarded as the promoter of neuronal outgrowth, and this subcluster was first defined to induce secretome which promoted adult rodent DRGN neurite outgrowth. The patient‐reported pain maps showed that synovitis and the pattern of knee pain were affected by the changes from SFs subsets of (early stage OA, pain) to SFs subsets (end‐stage OA, pain). Notably, subsets of SFs of early OA patients promote fibrosis and inflammation in OA, and neuronal growth to activate the nociceptive signal pathways.

The ‘non‐painful’ early‐stage OA SFs (early‐stage OA, no pain) subclusters with higher expression of early‐stage OA, no pain (gelsolin) relate to synovial hyperplasia. However, GSN was downregulated in end‐stage OA subclusters, suggesting that protective mechanisms against inflammation and oxidative damage are gradually lost during OA progression. Using the scRNA‐seq data of SFs from two OA patients, Wu et al. explored that Fibronectin1 (FN1),^88^ a vital component of ECM that has been applied to repair cartilage degeneration,^89^, ^90^ has a similar function of inhibiting matrix metalloproteases to subclusters of early‐stage OA, no pain. Cai et al. found SFs in state 5, with the gradually higher expression level of VCAM1 and TLRs, enriching ERBB signalling pathway, have a similar ability to release chemokines and pro‐inflammatory factors with early stage OA, pain subclusters. SFs in state 5 could transit to SFs in state 2 that highly expressed genes similar to end‐stage OA, and pain. Whilst enhanced stimulation may lead SFs ‘progenitors’ to state 2, however, a detailed transition process needs further exploration.^91^ Collectively, different synovial tissue phenotype of different degree of knee OA pain largely relates to the distinct genotypes of synovial fibroblast subclusters. In addition, synovitis may be predictive of faster rates of cartilage loss due to the crosstalk with releasing of cytokines and chemokines mediators by SFs.

With a pro‐inflammatory phenotype, subclusters of synovial macrophages may also accumulate synovial inflammation and pain in OA. (Figure 4A) Bone marrow‐derived monocytes migrate to the injured sites and further differentiate into M1‐like or M2‐like macrophages. They affected nociceptors‐induced pain response by secreting pro‐ or anti‐inflammatory cytokines and chemokines. Moreover, activated sensory neurons produce neuropeptides to modulate inflammation in a return.^83^, ^92^ Actually, M1 and M2 macrophages are just two polar extreme states reported in a number of studies, however, scRNA‐seq could allow for increased appreciation of the cellular diversity of macrophages. Recent studies have demonstrated that a special kind of barrier macrophage subclusters in the outer layer of healthy knee synovium that highly expressed genes such as CX3CR1, inhibit inflammation with a tight‐junction‐mediated shield for intra‐articular structures.^84^, ^93^ CX3CR1+ lining macrophages have already been observed in mice and shown to involve in forming a structural barrier with tight junctions to protect synovium against inflammation. Corresponding with the human equivalent of CX3CR1+ synovial‐resident lining macrophages, NUPR1+ cell subclusters were found that increased in OA patients, limiting the synovitis.^84^ Interestingly, macrophages from meniscal tissue displayed differently from murine barrier‐forming synovial macrophages.^94^ The expression of CX3CR1 is missing, though some specific genes expressed by barrier macrophages could be found.

Synovitis was found to be related to obesity, which was responsible for specific inflammatory phenotypes by bulk RNA‐seq. Owing to obesity, loading became an important hallmark to elevate OA, resulting in the secretion of pro‐inflammatory cytokine. Croft et al. discovered that SFs subsets in obese OA patients were differentially impacted by synovitis. ‘Activated fibroblasts’ and ‘immune cell recruiters’ notably maintained ECM and the activation of leukocytes respectively. The ‘immune cell recruiters’ subset with high expression of CHI3L1 mediates inflammation in obese OA patients. These subsets are localized in the sublining and lining layers of OA synovium tissue which can be distinguished by FOS (transcriptional regulators),^95^ whilst MYC is only present in normal weight patients in the lining layer.^96^

### 3.3 Meniscus

Meniscus was divided into three regions because of the density of blood vessels (red zone, red‐white zone and white zone). Cell types in meniscus include chondrocyte‐like morphology cells and fibroblast‐like cells, and the inner region contains chondrocyte‐like morphology cells whilst the outer region contains fibroblast‐like cells. When meniscus degenerates in OA progression, the peripheral vessels fade and the fibroblast‐like cells populated, followed by collagen fiber network reorganized and the chondrocyte‐like morphology cells differentiated into fibroblast‐like cells.

Meniscus degeneration is an important risk factor for knee OA and their relationship is complex.^97^ Increasing biomechanical stress in the joint implicated by the loss of meniscal function results in damage such as cartilage loss, eventually leading to symptoms of OA. However, the heterogeneous nature both in terms of structure and function of the meniscus significantly precludes a further mechanistic understanding of meniscus‐mediated OA that may provide important insights into the pathogenesis of OA.^98^ scRNA‐seq revealed the various cell types of the meniscus and how its degeneration contributes to the development of OA.^99^

**FIGURE 5:**
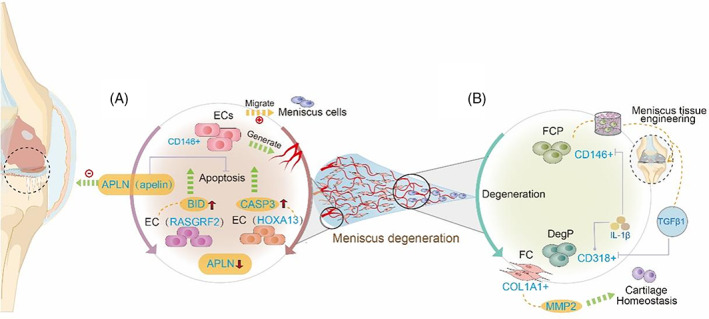
Applications of scRNA‐seq in meniscus. Single‐cell RNA sequencing reveals the progression of meniscus degeneration. (A) Apelin alleviates meniscus endothelial cell apoptosis in osteoarthritis. (B) FCPs and DegPs were identified as progenitors from healthy human meniscus cells and degenerated meniscus cells. FCs were correlated to the expression of MMP2 leading to cartilage destruction.

Sun et al. demonstrated the existence of progenitor cells–fibrochondrocyte progenitors (FCPs) with CD146 specifically highly expressed in healthy meniscus and degenerated meniscus progenitors (DegPs) marked with CD318 in OA meniscus.^100^ (Figure 5B) Isolated by FACS, the proportion of CD146+ primary human meniscus cells was near 2.7%, with the ability to differentiate into various cell lineages, including osteoblasts, and the clonogenicity was significantly higher than the CD146 group. CD318 increased rapidly at the end of the differentiation of FCPs to DegPs that were aberrant to the normal differentiation procedure. Thereby, DegPs may be a target for the recruitment of meniscus progenitor cells for tissue engineering.^101^ The two progenitor cell clusters can be affected by inflammatory mediators. IL‐1β decreased CD146+ progenitor cells but increased CD318+ cells, however, TGF‐β has the opposite ability. The specific cell clusters contributing to meniscus aberrant degeneration demonstrated a novel mechanism of meniscus degeneration. Another study showed FCs (fibrochondrocytes) populations in meniscus cells, with COL1A1 highly expressed, increased gelatinases such as MMP2, which may contribute to cartilage homeostasis in the physiological status.^102^, ^103^, ^104^

Among the meniscus zones, the red zone is rich in blood vessels. Vascularization of this region is the most active while the other two zones are avascular to a different degree.^105^ According to such heterogeneity in vascularity, scRNA‐seq discovered that endothelial cells played an important role in meniscus degeneration.^106^ ECs (endothelial cells in meniscus) progenitors (marked with CD146+) can not only generate vessels to maintain a blood supply, but also promote the migration of meniscus cells. (Figure 5A) Two ECs subclusters in OA patients were respectively marked with HOXA13 and RASGRF2. They expressed different intrinsic apoptosis factors, CASP3 and BID. And these two subclusters both showed downregulated expression of APLN (coding the receptor for apelin) resulting in enhanced apoptosis of endothelial cells that contributed to vessel fading in the red zone leading to meniscus degeneration. These data suggested APLN would be a potential target to prevent intrinsic apoptosis of ECs and inhibit meniscus degeneration of OA.

### 3.4 Subchondral bone

Beneath the hyaline cartilage and cement line, subchondral bone consists of the subchondral bone plate and subchondral bone trabecula. During OA progression, abnormal subchondral bone remodelling and angiogenesis in subchondral bone also contribute to cartilage destruction. The inhibition of osteoclast activity and remodelling processes associated with angiogenesis are the main reasons for the pathogenic mechanisms. Therefore, the microstructural and histopathological changes in subchondral bone are due to changes in the interactions amongst the multiple cell populations such as osteocytes, osteoblasts (OBs), osteoclasts, endothelial cells and so on.^107^, ^108^, ^109^ Activation of osteoblasts and endothelial cells began to activate H‐type vessels to invade in the subchondral bone, finally the new bone formed and osteophytes accumulated.

Accumulating studies perceived genetic networks to characterize the pathophysiologic progress of OA.^110^ RNA‐seq showed that IL11 (interleukin‐11) was identified as a robust OA risk gene in the regulating gene network.^111^ IL11 was proposed to associate with subchondral bone remodelling recently and up‐regulated in the cartilage leading to OA.^112^

**FIGURE 6:**
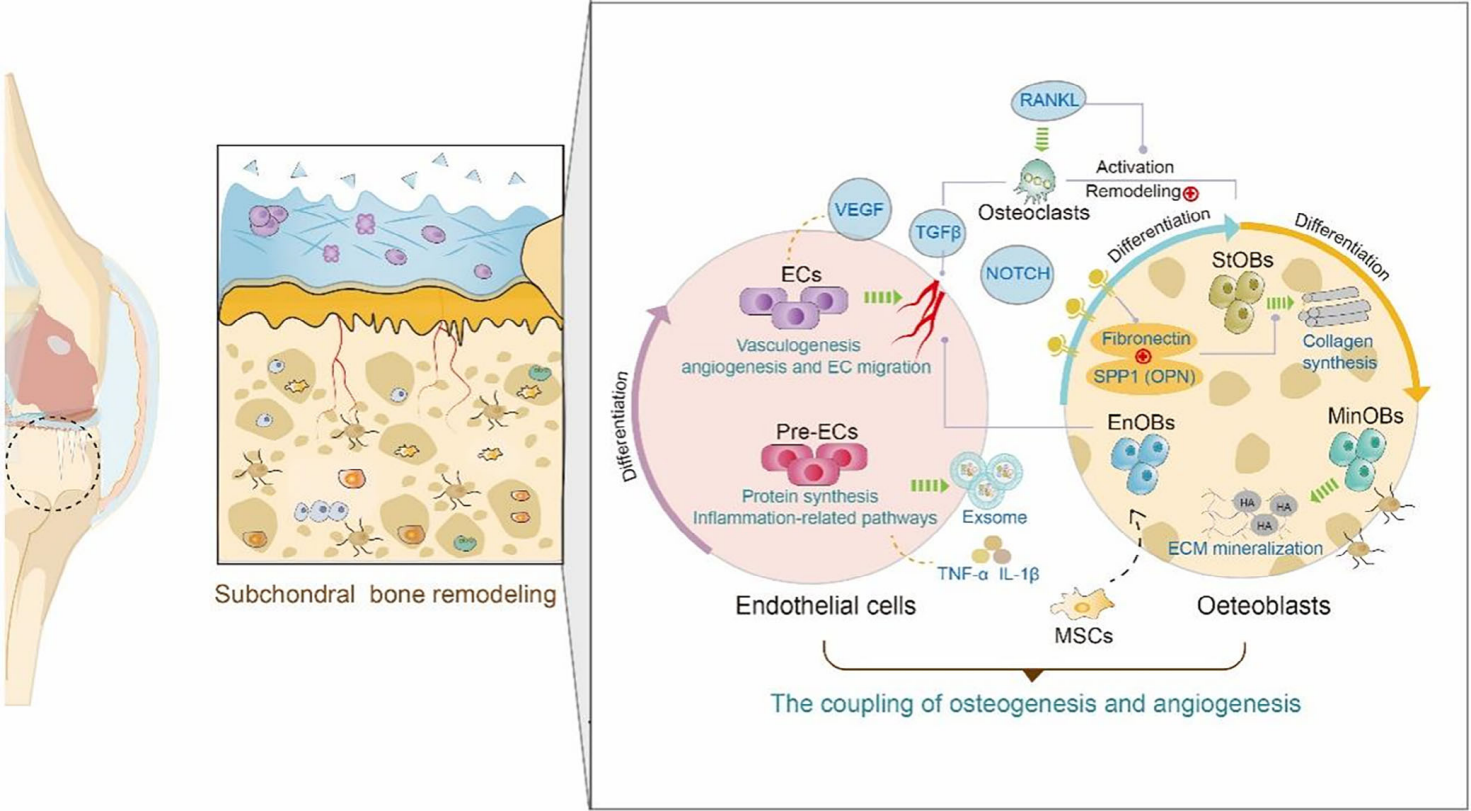
Application of scRNA‐seq in subchondral bone. Single‐cell RNA sequencing revealed the microenvironment changes in subchondral bone during the development of osteoarthritis. The coupling of osteogenesis and angiogenesis caused increased bone mineral density triggering sclerosis in subchondral bone.

Our group used scRNA‐seq to further identify the cell–cell communication networks in the subchondral bone.^113^ Notably, the coupling of osteogenesis and angiogenesis between OBs and ECs (endothelial cells in subchondral bone) may contribute to subchondral bone remodelling.^114^ (Figure 6) Previous evidence has shown that the crosstalk between OBs and type‐H ECs aggravates subchondral bone remodelling.^115^ As we noticed, Pre‐ECs (Precursor‐endothelial cells) are characterized by mediating inflammation pathways and exosome anabolism to participate in the crosstalk between chondrocytes and endothelial cells. ECs differentiated from Pre‐ECs could not only promote angiogenesis but also be coupled with OBs. Pre‐ECs can also promote the release of regulators for vascular development such as NOTCH, VEGF and TGFβ. Pre‐ECs showed higher capacity in promoting local bone formation by stimulating the proliferation and differentiation of MSCs or osteoprogenitors.^107^ Among them, TGFβ1 promoted by osteoclasts (activated by RANKL)^116^, ^117^ via Smad2/3 pathway to recruit osteoprogenitors negatively aggravating the turnover rate of subchondral bone remodelling and the formation of osteoid islets.^118^

We also demonstrated three phenotypes of OBs that comprehensively depicted their subtypes of them. Phenotypes changes from EnOBs (endothelial osteoblasts) to StOBs (stromal osteoblasts), and finally the spatial mineralization phenotypes MinOBs (mineralised osteoblasts) that relate to abnormal osteogenesis. EnOBs are tightly correlated with OBs proliferation and StOBs are associated with collagen fibril organization, assisted by OPN and fibronectin while MinOBs directly relate to ECM mineralization as the terminal state of bone formation leading to osteosclerosis in the end stage of OA.^119^ However, the osteoclasts were lost in the differentiation process during the single‐cell isolation progress, more detailed relationships of the microenvironment changes in OA subchondral bone should be elucidated in future studies.

scRNA‐seq helps to delineate the impact of changes in the patterning of various cells on pathological structures during OA progression, linking previous perceptions together to reveal consensual mechanisms. However, applications of scRNA‐seq in bone biology are just the beginning and recent research merely elucidated the potential cell subcluster targets, still lacking a strong indication of characterizations both in vitro and in vivo.

## 4 DISCOVERING TARGETS FOR OSTEOARTHRITIS

Based on the mechanisms elucidated in the previous context, scRNA‐seq has been conducive to discovering potential therapeutic targets, contributing new insights to the development of new therapeutics. Conventional omics analysis of joint tissues was used to identify candidate biomarkers or potential targets to elucidate the mechanisms of OA. However, previous analyses have merely focused on the associations of particular molecules or molecular changes with OA rate of progression, so far, rather than on the discovery of phenotypes.^120^ Advances in deeper phenotyping of cell types with scRNA‐seq enable researchers to further characterize OA phenotypes, contributing to the discovery of targetable mediators or pathways.^121^ In past decades, some of them are promising under clinical investigation in randomized controlled trials.^122^ Emerging therapies such as disease‐modifying OA drugs (DMOADs) include those targeting ECM‐degrading, promoting structural repair, limiting local low‐grade inflammation and inhibiting multiple signalling pathways.^123^, ^124^ Moreover, intense efforts have focused on tissue‐engineering, bone‐targeted delivery systems or composite biomaterials as non‐pharmaceutical treatments for OA therapies.^125^, ^126^, ^127^ Given a broad range of advantages, progenitors (similar function to MSCs) characterized by scRNA‐seq are one of the potential cell types to apply in osteogenesis tissue‐engineering construction.^80^

**FIGURE 7:**
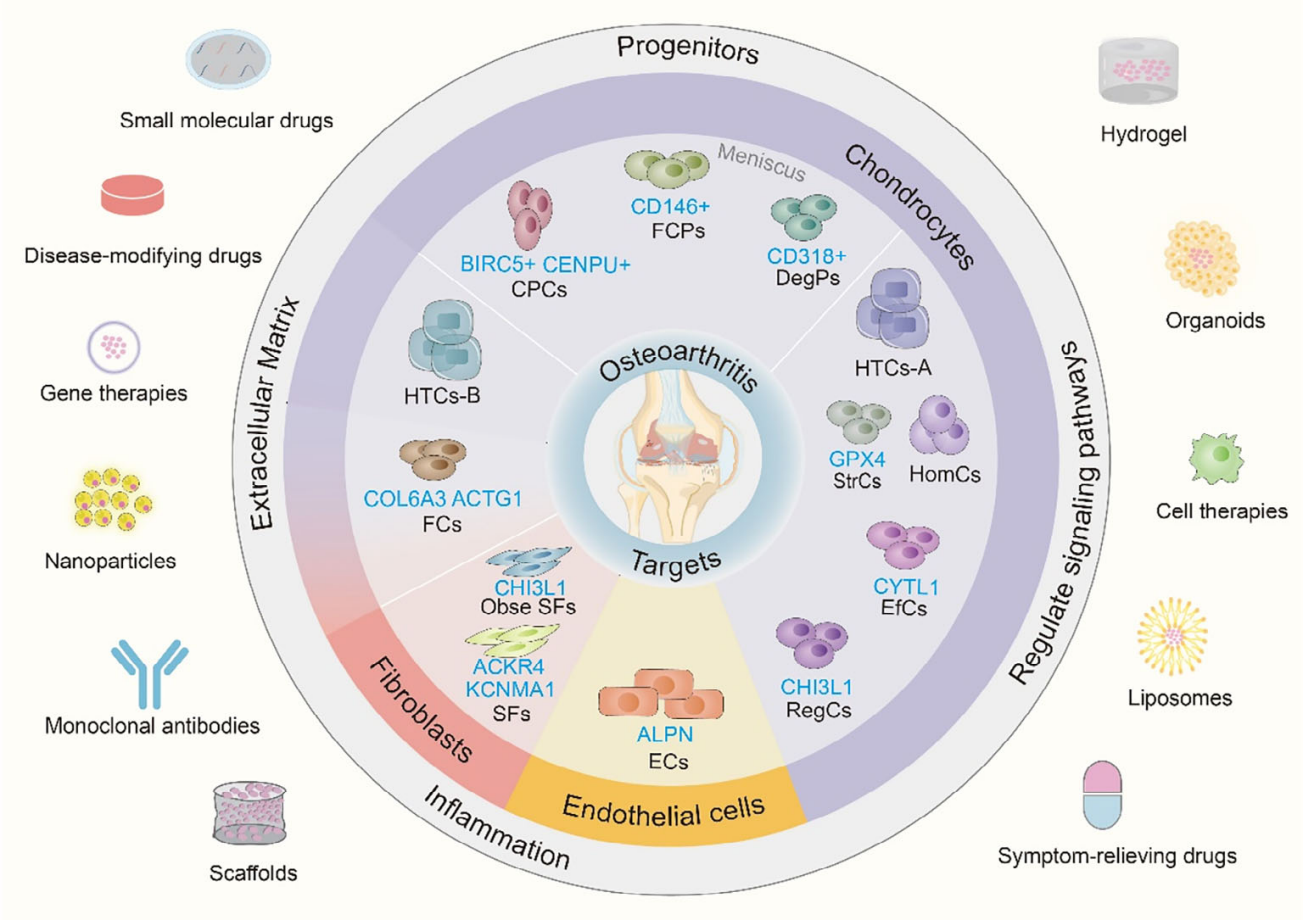
Key targets in OA discovered by scRNA‐seq. Currently, single‐cell RNA sequencing identified the potential targets of cell populations to facilitate the development of targeted treatments for OA.

To date, scRNA‐seq has provided new targets to regulate specific cell populations retarding OA progression and verified the feasibility from an unbiased perspective the same time, which supports to translation these findings into useful tools in clinical care. (Figure 7) For example, 14‐3‐3ε identified as a novel component of the TNFR2 complex exhibited a restricted expression pattern mainly in preHTCs (similar definition to previous studies)^53^ regulating chondrocyte metabolism.^128^ The expression level of miR‐17 is sensitive to superficial chondrocyte subpopulations in hyaline cartilage, C1 and C2, which limits swelling of the cartilage layer and maintains fluid pressurization.^104^ Both 14‐3‐3ε and miR‐17 are structurally important targets in maintaining cartilage homeostasis and prevention of OA. Hence in‐depth validation with scRNA‐seq compensates for a new strategy that targeted gene expression to regulate functional changes in cell subpopulations, and the discovery of these targets makes it possible to design better‐targeted therapeutics for OA.

### 4.1 Chondrocytes (articular cartilage)

Restoration of bone microenvironment integrating with seed cells is a promising approach to drive the development of bioactivity bone biomaterials. The seed cells used most widely in tissue engineering are chondrocytes and MSCs (or progenitors), facilitating to repair and regenerate of the damaged cartilage that restores the functional properties of the impaired joint.^129^ Up to now, autologous chondrocyte implantation (ACI) is the only approved cellular‐based therapy by Food and Drug Administration (FDA). Even if several randomized clinical trials provided evidence of the efficacy of ACI in the therapy of OA, long‐term follow‐up remains unknown.^130^ scRNA‐seq has identified CPCs (marked by BRC5 and CENPU)^53^ showed its potential for cartilage repair. Increasing studies showed that CPCs (progenitors/MSCs) as native (not culture‐expanded) joint‐resident^131^ could be augmented by intrinsic cartilage repair, migrating to the injury site. Meanwhile, CPCs appear to have advantageous immune privilege with major histocompatibility, which allows them to be implanted in an allogeneic context for OA therapeutics such as tissue‐engineering^132^ and cell therapies.^133^ Removing the mechanical load acting on the damaged cartilage could also provide a window of opportunity to re‐establish cartilage homeostasis.^134^ Apoptotic chondrocytes are common in the destruction of area. TRPV1, coupled with GPX4, had anti‐ferroptosis effect in chondrocytes in both human OA cartilage explants and DMM‐induced OA mice model.^76^ It would be a potential target to preserve cartilage anabolism and inhibit cartilage catabolism.

The cartilage ECM could not only withstand mechanical loads but also sense changes in the microenvironment during OA progression and transmit signals to the chondrocytes.^68^ Considering its indispensable value, inhibiting the fibrosis of FCs through the focal adhesion pathway targeting COL6A3 and ACTG1 will preserve the normal structure of ECM. Emerging paradigms that combine the rapidly expanding field of smart mechanoresponsive biomaterials and delivery systems with mechanical signal targets in OA treatments aim to prevent ECM degradation and promote reparative anabolic processes.^135^ HTC‐B subcluster in OA chondrocyte enriched for genes related to ECM organization, ossification and mineralization^53^, ^136^ might be an agent for ECM‐mimetic biomaterials.

A tiny switch of cell states through regulating many signalling pathways may affect the feature of different OA phenotypes. HTC‐A was important in cartilage development while HomCs regulated cell cycle and metabolic changes. Both cell clusters may provide new implications for constructing and developing effective OA drug delivery systems. The molecular processes underlying both homeostatic and pathologic circadian chondrocyte biology may not be uniform.^137^ Soul et al. have identified two patient subgroups that significantly dysregulated genes involved in circadian pathways, while one of the patient subgroups exhibited specific changes in the mechanosensitive nature of the molecular clock.^138^ CYTL1 relating to aging may decrease the population of E(f)Cs clusters involved in the regulation of chondrogenesis^139^ while CHI3L1, one gene expressed highly in RegCs, staging the progression of OA suggested the diagnosis function. Both of them could be biomarkers in the early stage of OA to supervise the progression. It was been discovered that the deletion of CYTL1 did not affect cartilage development, but the maintenance of cartilage homeostasis. CYTL1‐knocked‐out mice were more sensitive to OA cartilage destruction but showed normal endochondral ossification.^55^ On the other hand, many studies have found that CHI3L1 was significantly increased in OA patient joint tissues and IL‐1β‐induced cell models, which also increased in sclerotic OA subchondral bone, indicating the importance in the OA progression.^60^, ^140^

### 4.2 Chondrocyte (meniscus)

FCP (CD146+) as progenitors also shows the ability in meniscus repair to prevent OA in vivo. With regional differential chondrocyte phenotypes, chondrocyte‐like cells in the meniscus may gradually differentiate into fibroblast‐like cells in the degeneration. Meniscus tears typically do not spontaneously heal, therefore, to repair the avascular zones, recruiting and fibrochondrocytic differentiation of the resident cells is essential to restoring meniscus biomechanical function.^141^ Presently, the goal to prevent meniscus degeneration leading to OA is to restore the tissue structure whenever possible and a number of studies support those cell‐based strategies effectively against meniscus degeneration.^142^ Another way to protect against meniscus degeneration and OA development is to induce the meniscus cell phenotype. For instance, RNA‐seq discovered that the Mohawk (MKX) combination of TGF‐3 in decellularised meniscus scaffold induced MSC differentiation to a meniscus cell phenotype.^143^ Similarly, conversing the aberrant cellular state of DegP (CD318+), a special progenitor in OA meniscus, may potentially shift the unfavourable phenotype during the OA progression.

### 4.3 Endothelial cells (meniscus)

Different endothelial cell plays different roles in the heterogeneous microenvironment in joint tissues of OA. The meniscus is more susceptible to catabolic stimuli than articular cartilage, thereby, repairing the complex anisotropic phenotype concerning blood vessel development to disrupt OA development. ALPN has been regarded as a target in ECs subclusters of meniscus cells to antiapoptotic maintaining vascular activity in the red zone. On the other hand, pre‐ECs and EC are connected with pathological angiogenesis coupling with aberrant osteogenesis to accelerate OA progression in the subchondral bone.^144^ Considering the complex functions of these cell subtypes, future research should emphasise altering dynamic pathways to better understand the pathophysiology of OA and to design therapeutics translating into clinical benefit.

### 4.4 Fibroblasts (synovium)

The local low‐grade inflammation in OA is considered a potential therapeutic target as epidemiological studies have reported an association between synovitis, OA pain and structural damage.^82^, ^123^, ^124^ Scavenging pro‐inflammatory mediators (cytokines, chemokines, etc.) secreted by SFs and up‐regulating ACKR4 to decrease chondrocyte catabolism ensues may improve OA symptoms. Attentionally, SFs also promoted neuronal growth associated with OA pain and further consideration is needed to decide whether SFs will be the target to bring down sensitization of the central and peripheral nervous system.^85^, ^145^ In obese OA patients, CHI3L1 and INBHA secreted from SFs of obese and normal weight OA patients respectively, represented the inflammatory molecular endotype of OA SFs. They will be personalized targets for therapeutic intervention of different patient subgroups.

Developing treatments for OA remains challenging.^146^ With scRNA‐seq precisely considering heterogeneity underlying damaged tissue and the efficacy and safety profile of treatments, emerging therapeutics should aim at reducing the burden of symptoms as well as modifying the natural course of OA by slowing the biological processes.^124^ (Table 2).

**TABLE 2:** The candidates in different tissues of osteoarthritis joints discovered by scRNA‐seq.

Origins	Candidates	Biological process	Reference
Mice cartilage tissue	miR‐17	Sensitive to superficial chondrocyte subpopulations in hyaline cartilage Maintain cartilage homeostasis and prevention of OA	[104]
Synovial membranes and cartilaginous tissues from 20 patients knee OA	COL6A3 ACTG1	Prevent ECM degradation and promote reparative anabolic processes	[72]
Cartilage tissue from 10 OA patients	CPCs	Repair cartilage	[53]
	HTC‐B	Related to ECM organization, ossification and mineralization	
	HTC‐A	Cartilage development	
	HomCs	Regulated cell cycle and metabolic changes	
	CYTL1	Decrease the population of ECs (effector chondrocytes) clusters and involved in the regulation of chondrogenesis; Maintenance of cartilage homeostasis	[53]
Human articular cartilage samples	GPX4	Anti‐ferroptotic effect of Trpv1 in OA cartilage	[76]
Human meniscus samples	FCP	Progenitors in OA meniscus	[100]
	ALPN	Antiapoptotic maintaining vascular activity in the red‐zone of meniscus	[106]
Human subchondral bone samples	Pre‐ECs and EC	Pathological angiogenesis coupling with aberrant osteogenesis to accelerate OA progression in the subchondral bone	[113]
Synovial joint tissue	ACKR4	Decrease chondrocyte catabolism	[85]
Human synovial joint tissue	CHI3L1	Hang the inflammatory molecular endotype OA SFs; Staging the progression of OA suggested the diagnosis function	[95] [53]
	INBHA	Hanging the inflammatory molecular endotype OA SFs	[95]

## 5 EVALUATING THERAPEUTICS FOR OSTEOARTHRITIS

**FIGURE 8:**
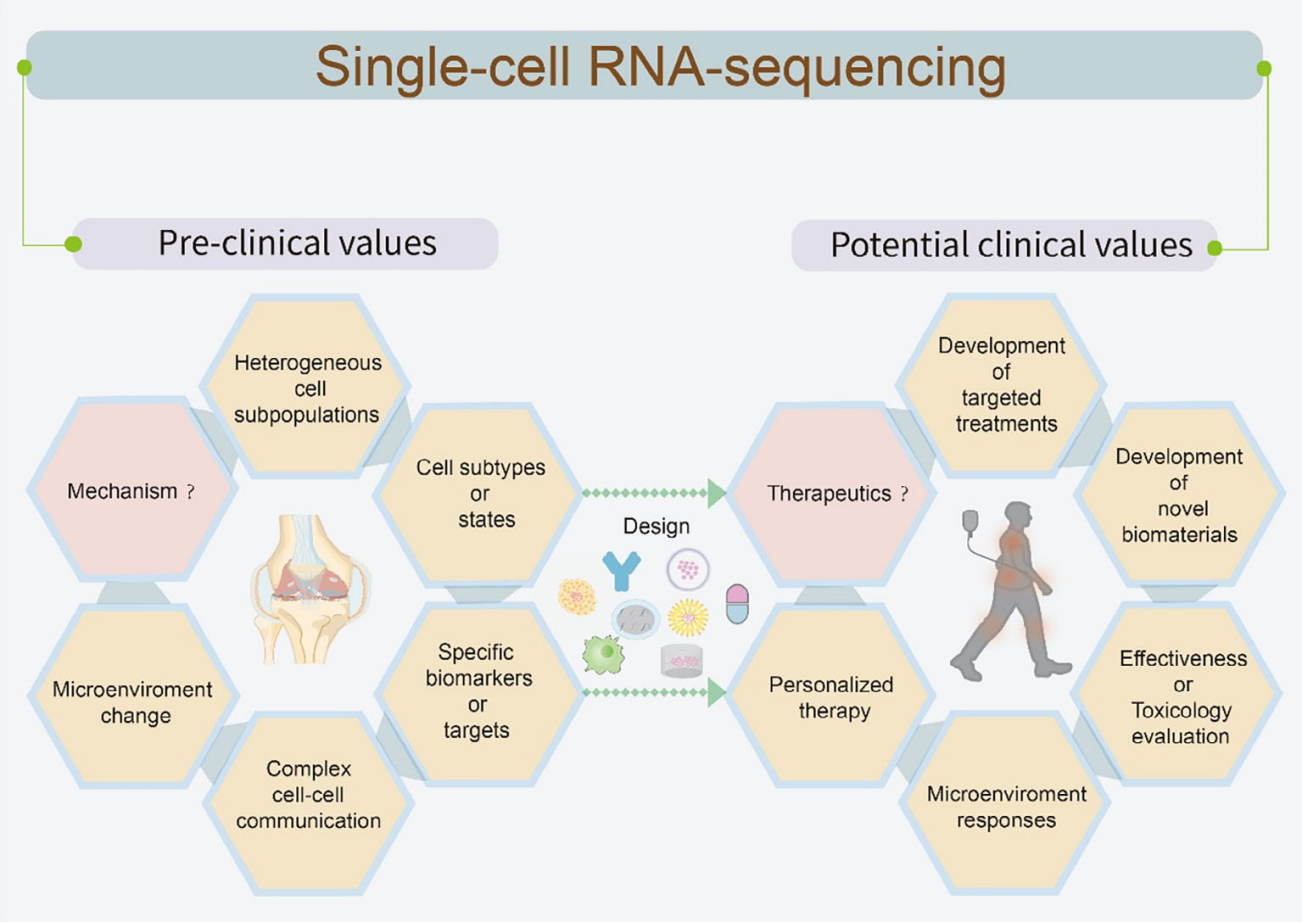
scRNA‐seq links potential clinical applications with pre‐clinical studies on OA. Single‐cell RNA sequencing is a promising technology in preclinical studies to identify potential molecular mechanisms or targets in OA and will be applied as a fundamental method in all aspects of OA clinical studies in the future.

scRNA‐seq is gradually used to evaluate the effectiveness or toxicity of therapeutics by discovering the microenvironment changes after accepting the objective treatments. Indeed, based on the mechanisms and targets revealed by scRNA‐seq to design therapy strategies, as a return, it can also reevaluate the most suitable to conduct in the pre‐clinical trials. (Figure 8) Patients responding non‐uniformly to treatments in health care remain a major challenge.^29^ With a broad range of genetic or environmental factors leading to different patient‐specific drug responses, cell‐to‐cell heterogeneity is increasingly considered as a basic contributor, precisely speaking, an endogenous driver of differential drug responses. The complex cell‐to‐cell heterogeneity indicates the demand for single‐cell techniques in precision medicine.

One promising single‐cell phenotyping tool with a massive scale (100,000 clones per experiment) for capturing phenotypic heterogeneity entails continuously observed over time microscopically.^147^ This tool has quantified the responses of single‐cell derived acute myeloid leukaemia clones for targeted therapy. Furthermore, single‐cell tools can be extended to decipher the higher‐level cellular architectures such as organoids.^148^ scRNA‐seq can also provide powerful personalized approaches enabling targeted therapy via currently available monoclonal antibodies or small‐molecule inhibitors. A case reported that employing scRNA‐seq successfully guided an altered therapeutic intervention on drug‐induced hypersensitivity syndrome/drug reaction with eosinophilia and systemic symptoms (DiHS/DRESS), which lacked animal models.^149^ Moreover, the resistance and prognosis of traditional treatments could be evaluated with scRNA‐seq. This practice is well established in the cancer field leading to the final aim of personalized therapy. Another study defines a roadmap integrated with scRNA‐seq data in the clinical trials (NCT04065789) and identifies resistance mechanisms of highly resistant multiple myeloma (MM) patients, facilitating the design of molecularly informed diagnostics and personalized therapy.^150^ Safety and effectiveness were the primary clinical endpoint. Using machine learning, scRNA‐seq has a large clinical value to predict patients' outcomes and evaluate the effectiveness of specific treatments, especially promising for biomaterials.^151^ scRNA‐seq enables to predict of disease outcome and characterization of Chimeric antigen receptor (CAR)‐T cell behaviour, therefore, it will become a standard tool for the clinical monitoring of CAR‐T cell therapies.^152^

Joint replacement surgery, the most common treatment method for OA therapy, is mainly focused on the late stage of the disease progression. The limited therapeutics hinder the available clinical options for patients. Meanwhile, there are growing requirements from the increasing occurrence of musculoskeletal disease in the aging population, demanding high‐quality bone grafts or tissue‐engineered biomaterials. However, the obstacle in the development of bioactive biomaterials and clinical applications is that the biological processes involved in osteoconduction capability lack a comprehensive explanation for further use. Recent developments in smart biomaterials give a chance to guide cell behaviours and responses when building the complex bone microenvironment. Thereby, detailed landscapes at a high resolution provided by scRNA‐seq will accelerate bone tissue engineering development.^153^ The complexity of degradation‐related cell populations around the implanted biomaterials in situ remains unclear.^154^ However, it is important to evaluate the biocompatibility of degradation‐related biomaterials and whether they have appropriate degradation rates or not.^114^, ^155^, ^156^ Huang et al. utilized mulberry silk to construct scaffolds and evaluated the macrophage clearance that controls the degradation of silk scaffolds in situ. This study identified macrophage subpopulations relating to material degradation, MD1, MD2, MD3 and the catabolic functions could change with the fibre density directly affected by MD2 and MD3 via MAPK1‐Stat3 signalling pathway.^157^ Guo et al. constructed hydroxyapatite (HA) scaffold together with ECM secreted by Wharton's jelly of the human umbilical cord (WJMSCs) to reveal the mechanism of osteogenesis process through the functional pathways such as cell proliferation and differentiation, ECM remodelling, hypoxia and inflammation responses in the microenvironment.^158^ According to these data, it is speculated that with chronological activation of signalling pathways such as TGF‐β, SOX9, PTHrP and BMP in the osteogenic lineage was related to endochondral ossification. In the late‐stage, endochondral ossification process was more likely to happen over intramembranous ossification, supported by the activation of Wnt signalling pathway and RANKL. However, the inflammatory response in HA scaffold was up‐regulated suggesting that balancing the osteogenic process of biomaterials and inflammatory microenvironments in cell subtypes would be a productive window. Together, Guo et al. highlight that the choice of intrinsic bioactive properties is important when modifying the traditionally applied HA scaffolds for tissue‐engineered bone, moreover, the balance of intervention timing decides the biological process for the bioactive materials. scRNA‐seq showed that subtypes can regulate the different responses to the biomaterials and this will provide a basis for modifying the biological properties of bone tissue engineering. Typical use to construct more bioactive material is applying bone organoids as an in vitro engineering model or therapeutics. Osteo‐callus organoids were verified to have specific gene expression patterns emulating the endochondral ossification process and scRNA‐seq also revealed their behaviours similar to the diverse cell compositions of the developmental process.^159^ With numerous functions, scRNA‐seq will be an essential tool for evaluating the complex biomaterials for bone therapy.

As mentioned before, interpatient heterogeneity in OA has an extraordinary impact on the devising of pharmacological strategies or tissue‐engineering treatments. Thus, evaluating the effectiveness of therapeutics amongst different patients allow for precise treatments of OA. To further understand how CPCs contribute to disease interpatient heterogeneity and compensate for the uncovered scRNA‐seq data in the previous study,^53^ Grandi et al. mapped both the pro‐regenerative and inflammatory cell populations based on the stratification of OA patients.^160^ In a low‐inflammation microenvironment, different subpopulations of CPCs have different regenerative potentials. It was reported in the study that CD24 could be a potential marker for CPCs subpopulations with higher regenerative capability. Additionally, the subcluster lost in OA, CPC I, was supposed to be progenitor cells, refining the definition of CPCs subpopulations to evaluate whether reintroduction of them could benefit cartilage repair in future OA treatments. scRNA‐seq data can also construct a model of the prognosis of patients suggesting the most promising therapeutic targets. On the basis of an integrated analysis of scRNA‐seq and bulk RNA‐seq, Shao et al. revealed the differentiation states of osteoclasts in osteosarcoma (OS) and their significant prognostic values. A nomogram was generated with both signatures of osteoclasts differentiation‐related genes (ODRGs) and clinicopathological parameters for clinical to validate it whether can predict OS patient outcomes with OS cohorts or not. Results of the differentiation of osteoclasts suggested their important role in prognosis.^161^ Similarly, before chemotherapy, it is rather challenging for researchers to recognize the chemoresistance of OS. Thereby, establishing an effective chemoresistance risk scoring model to evaluate chemoresistance in prechemotherapy can be accomplished by single‐cell sequencing technologies.^162^

Mapping genotypes to phenotypes is one of the diagnosis functions of scRNA‐seq and precisely typing different patients contributes to personalize therapy with specialized drug responses. A scRNA‐seq study confirmed the variable clinical symptoms in different OA patients.^4^ OA patients have been classified into four subtypes with distinct molecular signatures: C1: glycosaminoglycan metabolism disorder (typical clinical symptoms); C2: collagen metabolism disorder (osteophytes); C3: activated sensory neurons (perhaps neovascularization leading to joint pain); C4: inflammation (a narrowed joint space relating to inflammation). Contradicted traditional OA diagnosis, this work provided a precise diagnosis of OA and further evaluate outcomes of available therapeutics targeting the specific subpopulations. However, research on using scRNA‐seq technologies to evaluate therapeutics for OA remains to be developed.

To sum up, scRNA‐seq is expected to be a fundamental tool for translating safe and effective therapeutics for each patient into clinical reality rather than ‘one rule applied to all patients’. A powerful platform constructed by thousands of scRNA‐seq data will provide a roadmap to pinpoint drug reactions/biomaterial perturbations on the damaged tissues of each OA patient subset to establish personalized medicine strategies for OA.^163^

## 6 COMBINATORIAL SINGLE‐CELL MULTI‐OMICS TECHNOLOGIES IN OSTEOARTHRITIS

Investigating the disease progression with one‐dimensionally technology only provides a snapshot of the complex mechanisms. Consistent with a transformative new approach of NGS technologies, analysing genomics, epigenomics, spatial, proteomics and metabolomics at the cellular level provides an opportunity for scRNA‐seq to jointly learn across multiple types of data.^164^ Multiomics approaches should be combined to incorporate the complex interactions between regulations at multiple levels, along with longitudinal clinical data to provide a complete map of disease regulation.

In epigenomics, advances in single‐cell chromatin accessibility technologies have enhanced our ability to document cellular phenotypic variation. A robust method‐single‐cell accessible chromatin using sequencing (scATAC‐seq) revealing the landscape of cellular DNA regulatory variation that is systematically associated with specific trans‐factors and cis‐elements began to use in the regulation of hematopoiesis^165^ during human development.^40^ With specific barcode sequences such as the prokaryotic Tn5 transposase used in ATAC‐seq^40^ chromatin accessibility was simultaneously collected to select genomic DNA molecules. Therefore, co‐assaying thousands of single cells and identification of cis‐regulatory elements by a combinatorial indexing strategy will detect regulators associated with the expression of nearby genes. As chromatin accessibility is achieved, DNA‐methylation landscapes also provide new insights.^23^ To separate components of the genomic DNA fraction, before amplification, a sodium bisulfite treatment step captured the single‐cell DNA methylation patterns that can not be reflected by or inferred from RNA expression.^165^ Epigenetic and gene expression analysis has already been used and identified RWDD2B as a potential target of OA.^166^ This further indicates that the effects of epigenetic mechanisms on gene expression may be a major conduit that exerts OA genetic risk polymorphisms.^167^

In proteomics, high‐throughput and high‐content methods for protein analyses, are mainly mass spectrometry (MS). In metabolite analysis, matrix‐assisted laser desorption ionization (MALDI)‐MS has also been performed to characterize peptides. MALDI‐MS is the most applicable method in the rheumatology field and combined with imaging further allows the detection of peptides and proteins in situ.^168^ Recently, a format leveraging the precision of MS for flow cytometry has been developed.^169^ The fusion of the two technologies provides over 40 cellular parameters simultaneously, significantly augmenting the ability to evaluate complex cellular systems. Single‐cell mass cytometry not only investigated protein levels but also posttranslational modifications can be quantified to elucidate the functions of each protein and interactions between them. Proteomics has been used to discover the composition of cartilage or mineral density as an indicator of disease stage in OA.^18^, ^170^

Spatial omics emerged as a new frontier of rapid identification of cell types and spatial distributions.^171^ The spatial information is missing in scRNA‐seq, but spatial transcriptomics emerged to deal with the problem, which has also made the whole transcriptome or even epigenome measurable. Briefly, spatial distribution patterns are similar to immunofluorescence, therefore, training a machine learning model with multiplexed immunofluorescence imaging in spatial segmentation will broaden its applications in the future.

The integrated use of single‐cell technologies in cancer research has undoubtedly revolutionized the relationships across cellular modalities and holistic understanding of the biological characteristics of tumours.^172^ Matched scRNA‐seq/scATAC‐seq has uncovered relevant mechanisms that drive transcriptional programs in cancer cells of tumorigenesis in human ovarian and endometrial tumours.^173^ Single‐cell multi‐omics methods can also probe regulatory states and cell‐fate determination in aging and making it amenable to intervention.^174^ Using single‐cell whole‐genome sequencing to perform genome‐wide somatic single‐nucleotide variant (sSNV) in DNA from prefrontal cortical and hippocampal neurons showed somatic mutations were age‐related.^175^

Without the frontier methods, traditional multi‐omics were used to identify genes in biological processes of osteophyte development such as ERK1 and ERK2 cascade between chondrocytes from osteophytes and articular cartilage tissue from OA patients.^176^ However, the resolution is low to tell the gene expression in specific cell populations. By integrating single‐cell transcriptomics and mass cytometry methods, joint synovial tissues of RA patients have been identified with different inflammatory cell states. Inflammatory mediators such as IL6 expression mapped to THY1HLA‐DRA^+++++hi^ fibroblast populations were related to different cell states.^84^ In addition, single‐cell multi‐omics technologies have also been applied in OA research to further complement transcriptomics. A study has built the first articular cartilage proteomic cell atlas and identified two rare chondrocyte subclusters, inflammation‐amplifying‐A (Inf‐A) and inflammation‐amplifying‐D (Inf‐D). Inf‐A is related to recruit immune cells while Inf‐D with CD24 expressed may relate to the chronic inflammation in the end‐stage of OA. Interestingly, characterizing chondrocyte populations with the abundance of OA patients, the interactions between CPCs and Inf‐A or Inf‐D clusters in multifactorial systems drove the heterogeneity in different OA patients with different degrees of inflammation. Furthermore, the cell maps revealed by these multi‐omics methods allow for attenuating the negative effect of risk genes as an exploitable target for OA therapeutic interventions.

In summary, generation of single‐cell multi‐omics datasets has revealed many new insights into the biological process of musculoskeletal disease, but translating these big datasets into advanced diagnostic tools and feasible therapeutics is still a long way to put them into realized.^177^ One long‐existing challenge is how to address the sheer size of computational issues in scRNA‐seq. Furthermore, combined with the multi‐omics integration of two or more technologies, these issues will exacerbate the problem. From both an infrastructure and algorithmic view, challenges in designing computational methods and missing standard principles may limit our understanding of specific biological processes in cellular state changes because of transient cell states masking the underlying cell identity.^178^ Also, insights gained from single‐cell multi‐omics in the scope of genomic variation—specifically intronic variation remain doubtful due to lacking biological validation. Therefore, determining which biological signals should be considered confounders and the particular system deciding valid cell‐type differences is also a potential factor.^179^

## 7 CONCLUSION AND PERSPECTIVE

The booming combinatorial strategies and advances in sequencing platform such as microfluidics with low costs have empowered single‐cell sequencing technology to an exciting level. However, there are still many challenges involved in computational data, collating data analysis to biological progress and implementation in the realm of precision medicine during a single experiment.

One of the challenges in interpreting the data is assembling them into a patient‐centric context or a clinical problem. Collectively, multiple and iterative layers of individual patient complexity would be added when longitudinally evaluating biological samples. However, individual categorized treatments in precision medicine will be developed aided by computational and machine learning approaches. Then, considering the applications in recent research, underpowered studies with low sample sizes may miss true signals and are susceptible to producing false‐positive results. On the other hand, lacking a ‘gold‐standard’ data analysis pipeline miring reproducibility is a common problem in multi‐omics technologies. Building global data sharing repositories such as biobanks (e.g., osteoarthritis initiative etc.)^180^ with commonly accepted data may combat these challenges. Machine predictive analyses give access to integrate and compare results for OA researchers. scRNA‐seq technologies may become so widespread that biologists begin to routinely collect data in the future.

However, in bone biology, scRNA‐seq still has much shortness remaining to be solved. Bone tissue samples are hard to obtain not to mention the isolation problems, thereby, the best single‐cell protocols with high throughput per cell in case of missing important cell clusters. However, with the complex mix of heterogeneous cells, deciphering the sequencing data without the interference of MSCs needs to be consistent with the aim of the study. Currently, studies using scRNA‐seq to elucidate the mechanisms or find potential targets with the bioinformatic tools lack a common sense that the identified cell population should be recorded and even applied or characterized in later studies. This will help to build the whole cell atlas for the OA pathogenesis and classification of patients with discovered biomarkers by scRNA‐seq. The applications in evaluating the therapeutics are increasingly developing, and shortened in characterization in vivo and in vitro. With single‐cell multi‐omics technologies, better treatment for each patient will finally be invented.

In conclusion, we reviewed the recent applications of scRNA‐seq in OA. scRNA‐seq can not only reveal the mechanisms that different cells phenotype in damaged structures relating to OA progression, but also discovered targets through data analysis for better therapeutics construction. With increasingly applied in different problem solving, scRNA‐seq will contribute more resources to re‐examine the OA pathophysiology comprehensively and evaluate the safety and effectiveness of developing therapeutics in pre‐clinical research for OA.

## AUTHOR CONTRIBUTIONS

Yuyuan Gu and Yan Hu conceived, wrote, revised the manuscript and made the figure and table. Hao Zhang and Sicheng Wang revised the manuscript. Ke Xu and Jiacan Su reviewed, revised and edited the manuscript. All authors read and approved the final manuscript.

## CONFLICT OF INTEREST STATEMENT

The authors declare no potential conflicts of interest.

## Data Availability

Data availability is not applicable to this article as no new data were created or analyzed in this study.
